# Alveolar bone repair of rhesus monkeys by using BMP-2 gene and mesenchymal stem cells loaded three-dimensional printed bioglass scaffold

**DOI:** 10.1038/s41598-019-54551-x

**Published:** 2019-12-03

**Authors:** Liyan Wang, Weikang Xu, Yang Chen, Jingjing Wang

**Affiliations:** 1 National Engineering Research Center for Healthcare Devices, Guangdong Key Lab of Medical Electronic Instruments and Polymer Material Products, Guangdong Institute of Medical Instruments, Guangzhou, Guangdong 510500 China; 2 Department of Stomatology, Foshan Woman and Children’s Hospital, Foshan, Guangdong 528000 China; 3 Department of Orthopaedics, The First people’s Hospital of Foshan, Foshan, Guangdong 528000 China

**Keywords:** DNA and RNA, Implants

## Abstract

Over the past years, the study about bone tissue engineering in the field of regenerative medicine has been a main research topic. Using three-dimensional (3D) porous degradable scaffold complexed with mesenchymal stem cells (MSCs) and growth factor gene to improve bone tissue repair and regeneration has raised much interest. This study mainly evaluated the osteogenesis of alveolar bone defects of animal in the following experimental groups: sham-operated (SO), 3D printed bioglass (3D-BG), 3D-BG with BMP-2 gene loaded CS (3D-BG + BMP/CS) and 3D-BG with rhesus marrow bone MSCs and BMP/CS (3D-BG + BMP/CS + rBMSCs). Simulated human bone defect with critical size of 10 × 10 × 5 mm were established in quadrumana - rhesus monkeys, and in *vivo* osteogenesis was characterized by X-ray, micro-Computed Tomography (mCT) and history. Our results revealed that 3D-BG + rBMSCs + BMP/CS scaffold could improve bone healing best by showing its promote osteogenic properties *in vivo*. Considering the great bone repair capacity of 3D-BG + BMP/CS + rBMSCs in humanoid primate rhesus monkeys, it could be a promising therapeutic strategy for surgery trauma or accidents, especially for alveolar bones defects.

## Introduction

Regeneration of bone tissue depends on the osteoprogenitors’ host source and defect size^1,2^. Critical size defects won’t repair spontaneously and completely^1^. Moreover, alveolar bone defects caused by trauma, tumors or infections will compromise the osteoprogenitors’ host source. Thus dependence on only the local osteoprogenitors for bone repair and regeneration is impractical^2^. Autograft, allograft or biomaterials have been proposed for bone repair procedures. However, limited source and immunological rejection restrict the administration of autograft and allograft tissue replacement^3^. Tissue engineering strategies can overcome the drawbacks of autografts and allografts. Scaffold, cell and growth factor are the three important factors for tissue engineering approach^4^.

Bioglasses (BG) are artificial materials with good biocompatibility^5^. The Ca^2+^ could diffuses and deposits with the PO_4_^3−^ to SiO_2_ gel layer in biological fluid, and mineralizes gradually, thereby forming hydroxyapatite (HA) coating on the surface to achieve bonding with bone tissue. There are many methods including salting out, freeze-drying and particle filtration for preparing 3D-printed bioaglass (3D-BG) scaffolds. However, these methods cannot control the morphology and size of pores, porosity and overall attachment of scaffolds accurately^6^. And scaffolds prepared by conventional methods often fail to achieve desired mechanical strength. In recent years, 3D printing technology has been proposed. It can precisely control the structure of the aperture in mild environment, which could prepare scaffolds with more regular pore structure^7,8^. In this study, the 3D-BG scaffolds were prepared by 3D printing method as basic scaffolds.

Mesenchymal stem cells (MSCs) have the capacity to self-renew and differentiate into osteoblasts, which have drawn more attention to their clinical therapy of bone defect^9^. One of the critical challenges that need to be overcome is directing MSCs differentiation^10^. Bone morphogenetic protein-2 (BMP-2) are the most widely used growth factors for MSCs osteogenesis and promoting tissue regeneration^11,12^. However, short half-lives, ease of deactivation and high administration dosage limit the bone repair clinical applications of BMP-2^13^. Gene transfer technology including BMP-2 gene transfer into MSCs have been reported effective in promoting bone formation, which could avoid the above problems^14^.

Many kinds of animals including rodents, rabbits, dogs, sheep, and non-human primates have been used to perform buccal bone defect model^15^. The incisors of rodents continue to erupt with wear, and cementum and bone on the root’s surface are being remodeled constantly, which changes the position of teeth^16^. Moreover, the metabolism of periodontal tissues is rapid, and even if artificially caused by periodontal bone defects, spontaneous regeneration is often performed, which is difficult to establish a suitable periodontal bone defect model. Traditional animal models of periodontal bone defects are mostly established in small and medium-sized animals such as dogs and rabbits^17^. However, these animals have less overall similarity with humans, which affecting the effectiveness and reliability of the results. In this study, rhesus monkeys were selected as the experimental animals. Not only its species is close to humans’, but its anatomical and functional state of periodontal system is similar to humans’ too^18^.

The purpose of this study is to provide an active tissue engineering scaffold for repair of critical size bone defects. This material was constructed with a composite material of 3D-BG scaffold, MSCs and BMP-2 gene loaded nanoparticles (BMP/CS). The tissue engineering scaffold was implanted into the defect of alveolar bone of rhesus monkeys, and its osteogenesis ability was evaluated. X-ray, micro-Computed Tomography (mCT) and tissue section were employed for characterizing the bone repair ability.

## Results

### *In vitro* apatite forming ability of 3D-BG scaffolds

**Figure 1:**
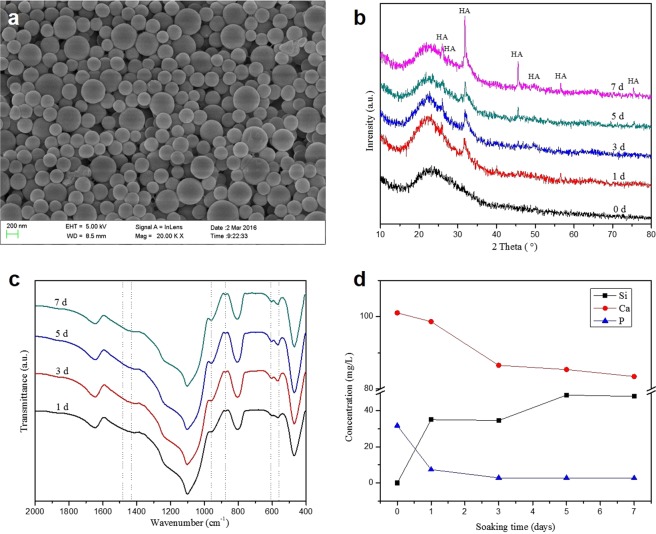
SEM micrographs, XRD patterns and FTIR spectra showed the morphology (**a**), physical structure (**b**) and chemical structure (**c**) of 3D-BG scaffold. *In vitro* ions concentration (Si, Ca and P) in SBF after soaking with 3D-BG scaffold for day 0.5, 1, 3, 5 and 7, which indicated the forming process of calcium phosphate on 3D-BG scaffold’s surface (**d**).

It can be observed that the fibers of 3D-BG scaffolds were formed by accumulating of BG microspheres, and good filamentous adhesions can be seen between the microspheres (Fig. 1a). FTIR spectra of the 3D-BG scaffolds showed only BG silica network (Fig. 1c).

**Figure 2:**
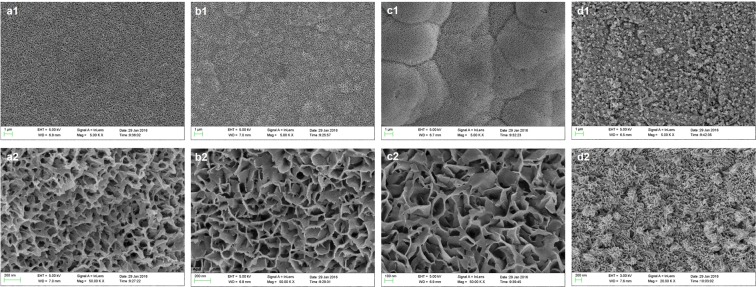
SEM micrograph of the 3D-BG scaffold after soaking in SBF for different times: 1 day (**a**), 3 days (**b**), 5 days (**c**) and 7 days (**d**), which showed the deposition process of apatite on the surface of 3D-BG scaffold.

The 3D-BG scaffolds were characterized by XRD, FTIR and SEM for evaluation of apatite formation ability after reaction in simulated body fluid (SBF). Characteristic diffraction peak of hydroxyapatite (HA) crystal was produced at 2θ = 26° and 32° after reaction in SBF (Fig. 1b). With the progress of the reaction, intensity of diffraction peak was increased continuously, and other characteristic peaks of HA appeared at day 7. P-O bending vibration peaks at 562 cm^−1^ and 603 cm^−1^, and P-O stretching vibration peak at 962 cm^−1^ and CO_3_2^−^ at 873 cm^−1^, 1430 cm^−1^ and 1480 cm^−1^ indicate that the crystalline hydroxyapatite carbonate (HAC) was formed at day 1 (Fig. 1c)^19^. The absorption peaks of scaffolds showed an enhanced trend at day 3, 5 and 7 of mineralization reaction. Figure 1d show the ion concentrations of silicon (Si), calcium (Ca) and phosphorus (P) changed. Concentration of Si ion increased rapidly at first, then increased slowly to the maximum, then decreased slightly and tended to be stable. Because there is no Si ion in SBF, the change of Si ion concentration in SBF can be regarded as a characterization method of the scaffold’s degradation. With prolongation of immersion time, the concentration of Ca and P ion increased at first and then decreased to stable. Morphology of the HA formed on the surface of the 3D-BG scaffolds was characterized by SEM (Fig. 2). Compared with accumulation of BG microspheres before reaction (Fig. 1a), honeycomb HCA was produced on the surface of scaffold at day 1 (Fig. 2a). Apatite deposited on the scaffold gradually, and larger scale like HA was formed at day 3 (Fig. 2b). Scale HA deposited on the surface and the thickness of apatite layer was increased at day 5 (Fig. 2c). Flower-like apatite was formed, and the surface was covered by it at day 7 (Fig. 2d).

**Figure 3:**
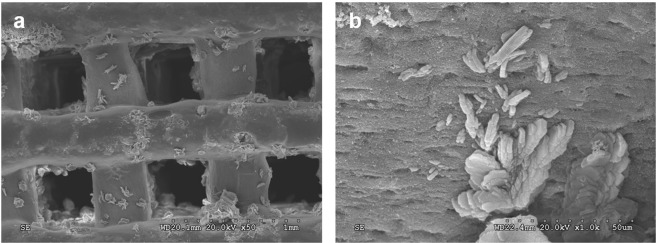
SEM micrograph of the 3D-BG scaffold finally applied in this study ((**a**): × 50, (**b**): × 1000).

After the *in vitro* apatite formation ability of 3D-BG scaffold was confirmed, a similar method was used to deposit apatite on the surface of scaffold, which enhanced the compressive strength of the scaffold (from 10.31 ± 1.21 MPa to 12.14 ± 1.42 MPa). It can be seen that after immersed in high concentration of phosphate buffer saline (PBS) for 3 days, the regular ordered macro porous structure of scaffold was still maintains (Fig. 3).

### Characterization of BMP/CS nanoparticles

**Figure 4:**
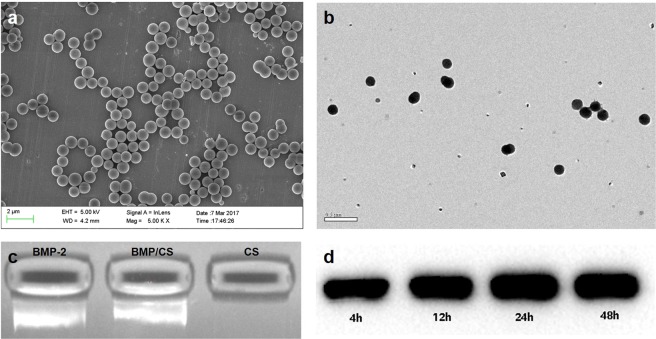
SEM (**a**) and TEM (**b**) images showed the morphology and adhesion property of BMP/CS. Gel block assay (**c**) showed the bound property between pcDNA3.1(+)-BMP-2 and CS. Western blot assay (**d**~**e**) showed the effectiveness of BMP/CS.

Morphology of BMP/CS nanoparticles was characterized by using SEM and TEM (Fig. 4). PcDNA3.1(+)-BMP-2 was complexed with CS and form regular spheres with a diameter of about 1000 nm (Fig. 4a). There is only a small amount of adhesion between spheres (Fig. 4b), which has little effect on their dispersion. The zeta potential of BMP/CS nanoparticles was about +6.5 mV, which was similar with others’ work^20^. The gel shift assay result showed that there was a bright band at the BMP/CS particle binding compared to the band shown by pcDNA3.1(+)-BMP-2 (Fig. 4c), indicating that pcDNA3.1(+)-BMP-2 and CS are partially but not fully bound. Western blot assay was performed to detect the expression of BMP-2 at protein level by adding CS solution and BMP/CS nanoparticles (Fig. 4d). The black band was detected by Western blot in the CS solution containing plasmid pcDNA3.1(+)-BMP-2. The expression of BMP-2 protein was positive, and the level increased with incubation time. The box was located in the rBMSCs with the addition of blank CS solution, and no BMP-2 protein was expressed.

### *In vitro* proliferation and osteogenic differentiation of rBMSCs on scaffolds

**Figure 5:**
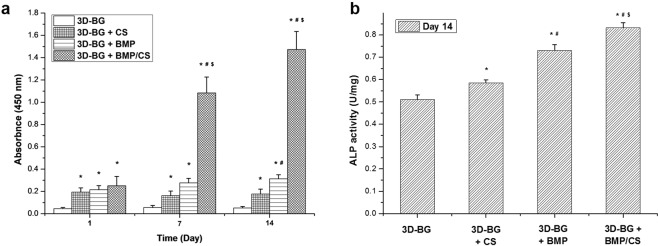
*In vitro* rBMSCs proliferation (**a**) and osteogenic differentiation (**b**) on 3D-BG, 3D-BG + BMP, 3D-BG + CS and 3D-BG + BMP/CS scaffolds (*P < 0.05 vs 3D-BG; #P < 0.05 vs 3D-BG + CS; $P < 0.05 vs 3D-BG + BMP).Compared with other groups, 3D-BG + BMP/CS could promote rBMSCs proliferation (**a**) and osteogenic differentiation signifificantly.

CCK-8 assay was used to characterize the proliferation of rBMSCs on 3D-BG, 3D-BG + BMP, 3D-BG + CS and 3D-BG + BMP/CS scaffolds. With the extension of culture time, the proliferation of rBMSCs on the four groups of scaffolds was similar, and the optical density (OD) values showed an increasing trend. And rBMSCs propagated rapidly in 3D-BG + BMP/CS group on day 7 and 14, and the number of cells was much higher than that of other groups (Fig. 5a).

In this study, rBMSCs on the scaffolds went through osteogenic differentiation. As a standard maker of osteogenic differentiation, alkaline phosphatase (ALP) was determined by pNPP assay (Fig. 5b). Positive ALP production was detected at Day 14. RBMSCs on the 3D-BG + BMP/CS displayed signifificant higher level of ALP secretion compared to other groups.

### *In vivo* osteogenesis

**Figure 6:**
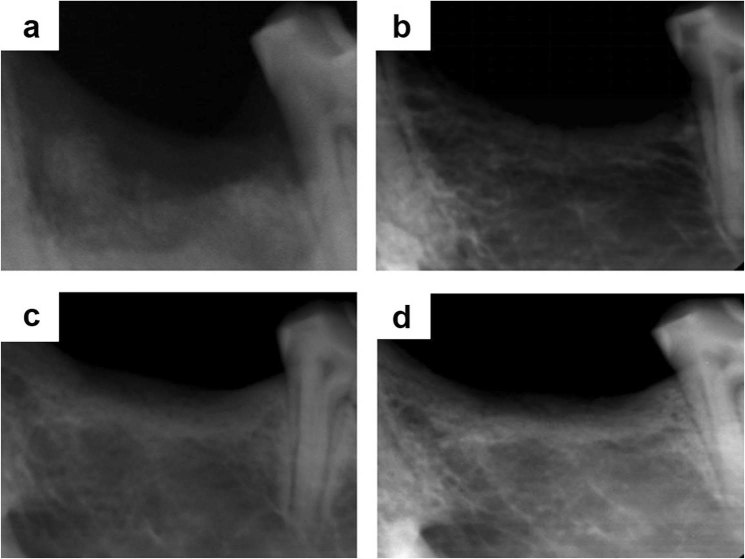
X-radiography showed the different implantation areas after 12w ((**a**): BC, (**b**): 3D-BG, (**c**): 3D-BG + BMP/CS, (**d**): 3D-BG + rBMSCs + BMP/CS).

After implantation for 12 weeks, X-ray photographs of rhesus monkey was shown in Fig. 6. The defect area of SO group was clearly visible, and no trabecular bone formation was observed. The bone structure was blurred and bone density in defect area was low (Fig. 6a). In 3D-BG group, the structure of new formated trabecular bone was observed. Compare with SO group, it can be observed that the arrangement of the of trabecular bone’s structures is much more regular, and the cortical bone is more continuous (Fig. 6b). In 3D-BG + BMP/CS group, a large number of new bone structures were formed. The trabecular bone structure is clearly visible, the mesh structure is evenly arranged, the cortical bone is relatively uniform and continuous. The alveolar ridge height is significantly increased (Fig. 6c). The bone defect area of 3D-BG + rBMSCs + BMP/CS group has no boundary with the surrounding normal bone tissue, the density is similar, the trabecular bone is evenly densely arranged in a network, the cortical bone is continuous, the height of the new alveolar ridge is increased, and the height of the normal alveolar ridge is very close (Fig. 6d).

**Figure 7:**
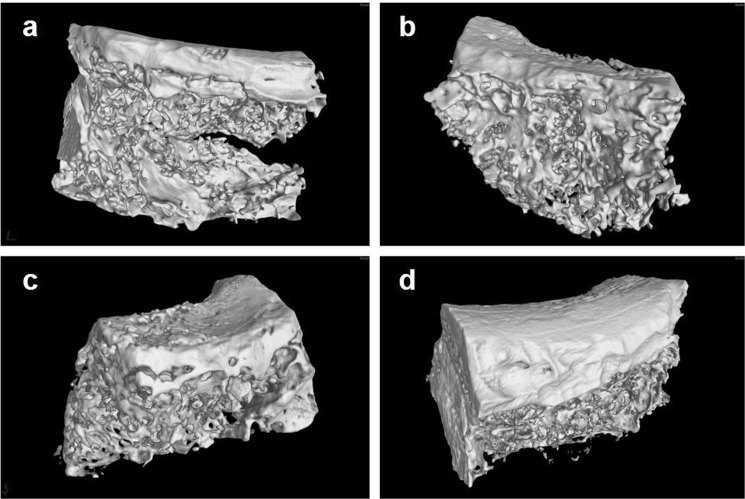
Micro-CT 3D reconstructions of implantation areas of rhesus monkey after 12 weeks, which showed the effect of bone regeneration ((**a**): SO, (**b**): 3D-BG, (**c**): 3D-BG + BMP/CS, (**d**): 3D-BG + rBMSCs + BMP/CS).

**Figure 8:**
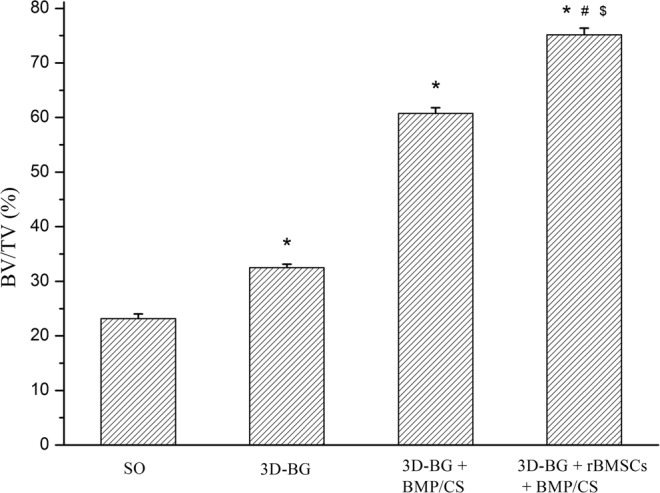
The bone volume of new alveolar bone in each group at 12-week post-operation (*P < 0.05 vs 3D-BG; ^#^P < 0.05 vs 3D-BG + CS; ^$^P < 0.05 vs 3D-BG + BMP).

Micro-CT 3D reconstruction model revealed new bone formation of each group (Fig. 7). Compared with SO groups, the trabecular of 3D-BG + rBMSCs + BMP/CS group was thicker, denser and more closely arranged (Fig. 7d). In SO group, the trabecula was disorganized and sparsely formed with cavities (Fig. 7a). The trabecular was clear in structure with large quantities in both 3D-BG and 3D-BG + BMP/CS group without cavities. The new bone was integrated with the surrounding bone without obvious boundary, and the bone defect healed well (Fig. 7b,c). By calculating, the BV/TV for each group was quantitatively determined. As shown by Fig. 8, P values between any two groups were <0.05, except that between 3D-BG and 3D-BG + BMP/CS groups. And regenerated bone of 3D-BG + rBMSCs + BMP/CS group was the maximum among all the groups.

**Figure 9:**
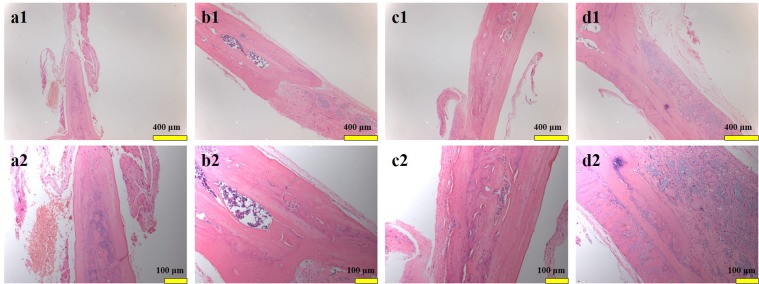
Hematoxylin and eosin (H&E) staining of bone tissues after implanted in rhesus monkeys for 12 weeks, which showed the effect of bone tissue regeneration; × 100 and × 400 ((**a**): BC, (**b**): 3D-BG, (**c**): 3D-BG + BMP/CS, (**d**): 3D-BG + rBMSCs + BMP/CS).

**Figure 10:**
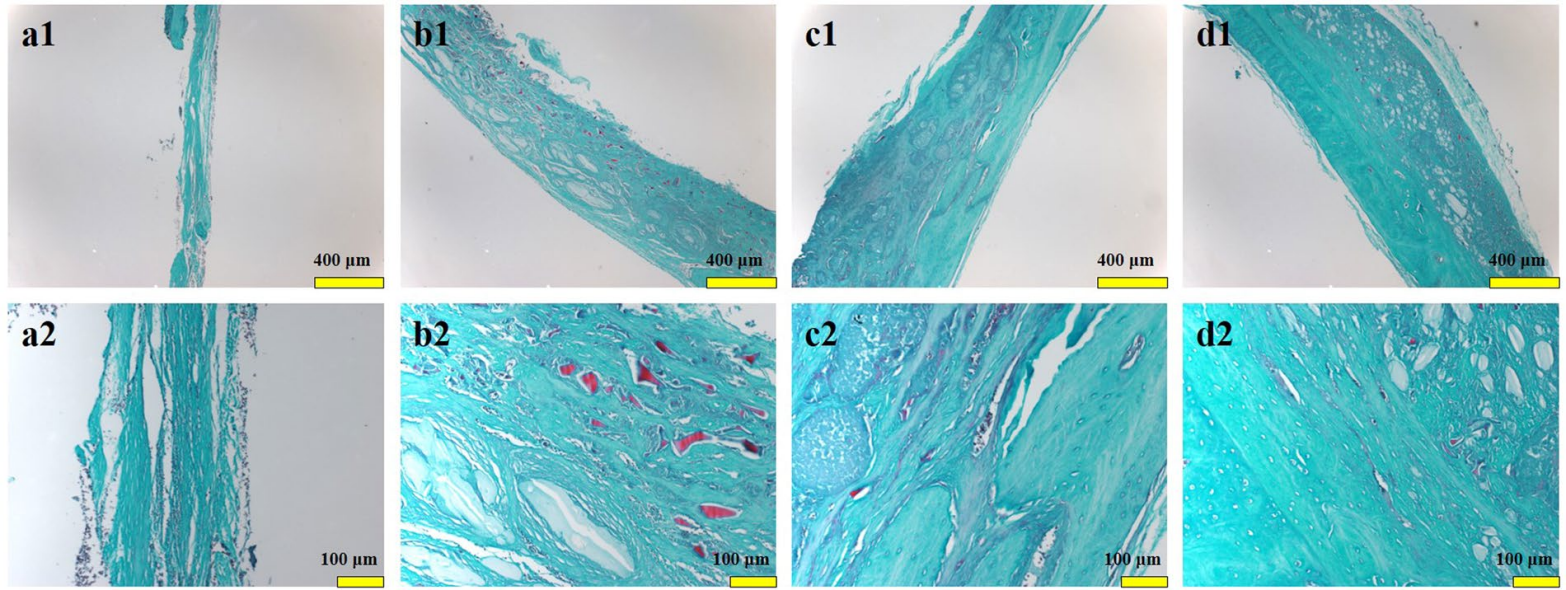
Safranine and fast green (S&F) staining of bone tissues after implanted in rhesus monkeys for 12 weeks, which showed the effect of bone tissue regeneration; ×100 and ×400 ((**a**): BC, (**b**): 3D-BG, (**c**): 3D-BG + BMP/CS, (**d**): 3D-BG + rBMSCs + BMP/CS).

12 weeks after implantation, histomorphological observation of rhesus monkey was shown in Figs. 9 and 10. There were no obvious signs of osteogenesis in bone defect of SO group, and there were some inflammatory cells infiltrating around (Figs. 9 and 10a). 3D-BG Group still has undegraded scaffolds, new bone tissue, and massive connective tissue in bone defect (Figs. 9 and 10b). 3D-BG + BMP/CS group has a small amount of undegraded scaffolds, surrounded by bone-like tissue around the unabsorbed scaffold material (Figs. 9 and 10c). The scaffolds in 3D-BG + rBMSCs + BMP/CS group were all absorbed, and a large number of new bones and new blood vessels were formed. The new bone surface can be seen with large active osteoblasts, with new bone marrow cavity formation, regular bone plate arrangement, and more mature bone tissue in some areas (Figs. 9 and 10d).

## Discussion

In this study, 3D-BG based scaffold was used to repair the alveolar bone defect of primate rhesus monkeys, which explore a new path for bone and tooth tissue repair. The genetically modified material constructed by combining rBMSCs and BMP/CS to 3D-BG scaffold, which is a novel bone repair material with high biological activity and gene regulation characteristics, and has not been reported in the existing research.

Alveolar bone defect with 10 × 10 × 5 mm was established as an acute defect model. There are two kinds of animal periodontal defect model: chronic and acute defect model^21,22^. The chronic defect model is the destruction of periodontal tissue caused by formation or enhancement of plaque stimulation by placing ligature wire around the teeth and quantitative injection of lipopolysaccharide (LPS) into gingival sulcus on a daily basis. Because of long time of forming defect model and the difficulty of homogenization, it is difficult to make the experimental statistics in later stage. In the process of making chronic defects, inflammation often involves gingival tissue, resulting in membrane gingival problems such as gingival redness, swelling or retraction, which affect the stability of wound suture, but is not conducive to the control and effect of regeneration experiment^23^. Acute defect model is to remove periodontal tissue including alveolar bone, cementum and periodontal ligament and make a model similar to the shape of clinical periodontal defect. The fabrication process is faster, and relatively unified shape and size can be obtained. Acute defects are similar to periodontal defects removed by root surface debridement, most of which are caused by chronic inflammation.

Scaffold could provide 3D niche for cells residence and tissue regeneration, which is an essential component in tissue engineering^24,25^. With the development of materials science, BG has been widely used in medical treatment^26^. In recent years, due to its unique inorganic amorphous structure, biological activity and bone-binding properties, it is considered to be an excellent bone and tooth restoration material^27^. 3D-BG scaffold has multi-layered holes structure and a functional supporting surface. Compared to other 3D polymer/BG scaffolds also using BMSCs reported, the size and shape and pore structure of 3D printed scaffolds can be controlled by the selection of different diameter needles, different fiber spacing and miscut displacement settings, which can meet various shapes of bone defect repair and reducing the impact of random structures^28^.

3D-BG scaffold was mineralized using high-concentration PBS to deposit a mineral layer on the surface before application (Fig. 3). It was believed that active ions released from BG could promote the proliferation and differentiation ability of MSCs, promote expression of genes and production of growth factors, and promote tissue regeneration^29^. The change of ion concentration in SBF is related to ion loss caused by material-induced apatite deposition. When scaffold was immersed in SBF, the Ca on scaffold surface dissolved quickly and then exchanges with H^+^ in solution. On the one hand, the non-bridged oxygen interacting with Ca in BG combines with exchanged H^+^ to form silicon-rich Si-OH gel layer on the surface. On the other hand, due to the continuous exchange of H^+^ in SBF, the content of OH^−^ increased gradually. Near the surface of BG in SBF, the concentration of Ca^2+^ and OH^−^ reaches supersaturation and interacts with PO_4_^−^ to form HA mineralized layer. At the beginning of the mineralization reaction, the release rate of Ca^2+^ was faster than that of apatite deposition, so the concentration of Ca^2+^ in SBF was higher at day 1, and then decreased and reached equilibrium slowly. However, the release rate of P ion from BG was slower than that of apatite forming at the beginning, so concentration of P ion in SBF decreased all the time. Concentration of Si ion in SBF has been increased continuously because SiO_3_^2−^ dissolved from BG, and Si ion has never participated in apatite deposition. With the prolongation of reaction time, the Si-rich gel was formed as a layer on the surface of BG and the deposited HA hindered the dissolution of internal ions gradually. So the release rate of BG ions slowed down. Finally, the whole mineralization system reached dynamic equilibrium^30^. The XRD, FTIR, ICP-AES and SEM results show that 3D-BG scaffolds have good apatite forming activity *in vitro*, and the existence of a small amount of PVA did not affect the biological activity of BG (Figs. 1 and 2). The 3D-BG scaffold has a pore size of about 0.5 × 0.5 mm (Fig. 3), which can support rBMSCs proliferation and osteogenic differentiation *in vitro* (Fig. 5), has good biocompatibility *in vivo*, has no adverse irritant response to animals, and can promote bone regeneration (Figs. 6 and 10).

Using growth factors to stimulate osteogenic differentiation of MSCs adhered on scaffolds, and improving bone tissue regeneration is a mainstream strategy for bone repair and regeneration^31^. However, growth factors possess short half-lives *in vivo* and also need high administration dosage^13^. By introducing gene transfection into bone tissue engineering, the above problems can be solved. It has been reported that BG combined with BMP2 gene could enhance bone formation ability in animals models the treatment of bone defects effectively^32–34^. The advantages of bioglass and gene loaded chitosan nanoparticles were combined, and 3D-BG + BMP/CS composite scaffolds were constructed for bone repair in this study. BMP/CS nanoparticles solution was added to 3D-BG scaffold before application. The CS and plasmid deoxyribonucleic acid (DNA) can be combined by electrostatic action to obtain BMP/CS spherical nanoparticles, and plasmid DNA is blocked partly (Fig. 3). As an ideal non-viral gene vector, CS has no potential infection risk and is not easy to cause immune response. CS and CS based polymer could complexed with nucleic acids including BMP2’s to protect them from enzyme degradation, and improve their transfection rate^35–39^. The addition of CS solution or BMP-2 solution to the scaffolds could significantly promote the proliferation of rBMSCs. However, the addition of BMP/CS solution promote the proliferation most (Fig. 4), which is consistent with others’ research^40^. After rBMSCs were seeded into 3D-BG + BMP/CS, BMP/CS nanoparticles contacted with rBMSCs and part of DNA were transfected and secreted BMP-2 protein, which would promote rBMSCs proliferation paracrinely/autocrinely^40^. In fact, our team’s previous work has shown that BG can promote gene transfection efficiency of CS/DNA nanospheres *in vitro*, the results now are consistent with that of previous^41^. The application of CS/DNA nanospheres has advantages *in vitro* DNA transfection research and data have shown their usefulness for gene delivery^42^. CS excels in enhancing the transport of DNA across the cell membrane through interaction with specific membrane receptors, which can elicit selected cell behavior including receptor-mediated endocytosis^43,44^. The molecular weight (Mw) and deacetylation degree (DDA) of CS, as well as the ratio of nitrogen to phosphorus (N/P), zeta potential and size of CS/DNA nanospheres could effect the transfection efficiency of DNA. The BMP/CS nanospheres synthetized in this study (N/P = 10, DDA = 92%, Mw = 10 KDa, diameter = 1,000 nm, zeta potential = 6.5 mV) might have good transfection effect according to the references^44,45^.

3D-BG, 3D-BG + BMP/CS and 3D-BG + BMP/CS + rBMSCs scaffolds were used as implants. Compared with 3D-BG scaffold, 3D-BG + BMP/CS scaffold could better promote the regeneration of bone defect, and promote degradation of scaffolds *in vivo* (Figs. 6 and 10). The reason may be that during the implantation of 3D-BG scaffold, K^+^, Ca^2+^ and PO_4_^3−^ are continuously dissolved, which affects the pH value of internal environment with the prolongation of time, which is not conducive to the proliferation of rBMSCs^46^. The combination of acidic CS and alkaline BG can adjust the pH value in environment, which is better for supporting cell proliferation^47,48^. In all experimental groups, 3D-BG + BMP/CS + rBMSCs group had the best bone repair regeneration and minimal scaffold residue. And regular lamellar structures were formed, and the new bone structure gradually matures and resembles the normal bone structure. BMP/CS attached to 3D-BG scaffold may stimulate and recruit surrounding MSCs to bone defect area, induce extracellular matrix mineralization of MSCs and pre-osteoblasts, produce BMP-2 protein and may increase osteocalcin and alkaline phosphatase expression^49^. At the same time, the produced BMP-2 induces MSCs to differentiate into osteoblasts and release into surrounding tissues, and all cells differentiated into osteoblasts may further release BMP-2 to promote bone regeneration. This is mainly due to the preloading of MSCs. rBMSCs mainly derived from bone marrow, which hold great promise for bone repair and regeneration owing to less invasive method^50^. These cells possess high proliferation and osteogenic differentiation ability that can grow on biomaterials^51^. Moreover, it has been reported that they had more similar biologic properties than human bone marrow MSCs^52^. Therapeutic efficacy of rBMSCs has also been evaluated and approved in regenerative medicine^53^. Compared with 3D-BG + BMP/CS group, rBMSCs preloaded on scaffold may reduce time to stimulate, recruit and induce differentiation of MSCs, which make scaffold degradation rate and bone formation speed match better.

## Methods

### Fabrication and characterization of 3D-BG scaffolds

3D-BG porous scaffold was prepared by 3D printing fiber deposition technology with BG microspheres (60% SiO_2_-36% CaO-4% P_2_O_5_, 350 nm in diameter, which was kindly provided by South China University of Technology) as printing raw materials and PVA as a binder. The scaffolds were prepared according to previously published literatures^54^. Briefly, 3D printing was performed by a 3D-Bioprinter (Envision TEC Gmb H, Germany) at room temperature to construct a cuboid BG scaffold of 10 × 7 × 5 mm with pore size of 500 um. The 3D-printed scaffold was then immersed into high concentration of PBS to form phosphate.

*In vitro* HA forming ability of 3D-BG scaffold was evaluated in SBF for 1, 3, 5 and 7 days, respectively. The morphology of the scaffold was observed by scanning electronic microscopy (SEM, Tescan Mira XMU, Tescan USA Inc., USA). Imaging was performed by sputter coating with gold, and a lens detector with a 5-kV acceleration voltage was used at calibrated magnifications. Phase composition of scaffolds was tested by X-ray diffraction analyzer (XRD, D8 Advance, Bruker, Germany). The scanning range of Cu target Kα ray (λ = 0.154 nm, tube voltage 40 kV, tube current 100 mA, step size 1°) is 10 to 80°. Surface groups of scaffolds were identified by Fourier transform infrared spectroscopy (FTIR, Vector 33, Bruker, Germany). Ions concentration in SBF was measured by Inductively coupled plasma-atomic emission spectrometry (ICP-AES, IRIS 1000, Thermo Elemental, USA).

### Preparation and characterization of BMP/CS nanoparticles

Chemical reagents of this study were purchased from Sigma-Aldrich. 10 g chitosan (CS, Mw: 10 KDa, DDA: 92%) was dissolved in 360 ml 1-methyl-2-pyrrolidone (NMP) containing NaOH and CH_3_I, followed by precipitation, dissolution, dialysis and lyophilization to obtain the product. A solution containing BMP-2 gene (pcDNA3.1 (+) - BMP-2, Guangzhou Chuangsai Biomedical material Co., Ltd., China) and above product, which their mass ratio of 1:6 was mixed, shaken vigorously (2500 rpm, 30 s) and placed in water bath (37 °C, 30 min). Then BMP/CS nanoparticles solution with N/P value of 10 was obtained. The Morphology of BMP/CS was characterized using SEM and transmission electron microscopy (TEM, JEM-2010, Japan). Electrophoretic mobility shift assay were run on 4% native gels with 1 × Tris–glycine buffer (pH 8.3) at 4 °C under 200 V constant voltage for 15 min. The gels were scanned using a Typhoon 9140 PhosphorImager (GE Healthcare). Western blot was used to detect the expression of BMP-2 level from rBMSCs by adding CS solution and BMP/CS separately. Western blotting was conducted using a Western blot kit in accordance with the manufacturer’s protocol (Zymed Laboratories, Invitrogen, CA). The mouse anti-human type I collagen monoclonal antibody (Sigma), the mouse anti-human type III collagen monoclonal antibody (Chemicon, Temecula, CA), and the mouse anti-human tenascin-C monoclonal antibody (Chemicon) were used as primary antibodies in this study.

### Extracting and culturing of rBMSCs

Healthy adult female rhesus monkey (7 years old, 3–5 kg) were selected for iliac bone marrow puncture. About 10 ml marrow was extracted and then cultured in DMEM/F12 medium with 10% fetal bovine serum and 1% penicillin-streptomycin double antibiotic (37 °C, 5% CO_2_). The cell culture medium was replaced every 2 days, and the cells were passaged every 3 days. After 1 week, the culture medium was replaced every 3 days. The fourth and fifth generations of the cells were spindle-like, arranged tightly, and grew in radial or whirlpool colonies, which were collected for preparing the tissue-engineered bone. Before rBMSCs were officially used for research, they were identified as having the ability of osteogenic differentiation (Supplementary Fig. S1).

### Preparation of experimental materials

The experimental groups were divided into different groups, namely sham-operated group (SO), 3D-BG, 3D-BG with BMP-2 gene (3D-BG + BMP), 3D-BG with CS (3D-BG + CS), 3D-BG with BMP-2 gene loaded CS (3D-BG + BMP/CS) and 3D-BG with rhesus marrow bone MSCs and BMP/CS (3D-BG + BMP/CS + rBMSCs). According to the size of materials, concentration of rBMSCs was adjusted so that each scaffold was seeded with 1 × 10^6^ cells. Infiltrating 3D-BG scaffolds with physiological saline (PS) before cells and other components were complexed. 3D-BG group, 220 μl of PS was added. 3D-BG + BMP group, 200 μl of BMP-2 gene (0.5 mg/ml) and 20 μl PS mixed solution was added. 3D-BG + CS group, 200 μl of CS (0.8 mg/ml) and 20 μl PS mixed solution was added. 3D-BG + BMP/CS group, 220 μl of BMP/CS solution (0.8 mg/ml) was added. 3D-BG + rBMSCs + BMP/CS group, 220 μl of BMP/CS solution (0.8 mg/ml) was added, followed by addition of cells suspension.

### *In vitro* rBMSCs proliferation and osteogenic differentiation on scaffolds

3D-BG, 3D-BG + BMP, 3D-BG + CS and 3D-BG + BMP/CS scaffolds (Φ5 × 2 mm) were used to study the *in vitro* cells proliferation property. 100 μl of cell suspension (1 × 10^6^ cells/well) were seeded onto the scaffolds. The scaffolds were left in the humidified incubator for 2 h to allow cells to attach on them, and then 400 μl of culture medium was added to each scaffold.

The scaffolds with cells were cultured for 1, 7 and 14 days (37 °C, 5% CO_2_). At various periods of time, 100 ul of fresh medium containing Cell Counting Kit 8 reagent (CCK-8, Dojindo, Japan) was added to each well and incubated for another 1 h at 37 °C. Cells seeded on bare Tissue culture plates (TCPS, no involvement of scaffolds) in the cultural media were used as control. The supernatants of each sample were aspirated and the absorbance was measured at 450 nm using a microplate reader (Epoch R, BioTek, USA).

Osteogenic differentiation was performed by incubating MSC laden scaffolds into osteogenic media (DMEM supplemented with 10% FBS, 100 nM dexamethasone, 50 mg/ml ascorbic acid, and 10 mM glycerophosphate) for 14 days. Cells that had been prewashed with PBS were lysed in 0.5 mL PBS containing 0.1 M glycine, 1 mM MgCl_2_, and 0.05% Triton X-100. The lysate solution was incubated with p-nitrophenyl phosphate solution at 37 °C for 30 min and subjected to a spectrophotometer on which the absorbance at 405 nm was measured and recorded to indicate ALP concentration. Total protein concentration was determined by BCA kit. ALP activity was calculated as the ratio of ALP concentration to total protein concentration

### Animal treatment and experimental design

All experimental protocols were approved by Ethics Committee on Experimental Animals of South China University of Technology. The animal experiments were comply with the ARRIVE guidelines and were carried out in accordance with the U.K. Animals (Scientific Procedures) Act, 1986 and associated guidelines.

Female rhesus monkeys (10-15 years old, 5–7 kg, n = 4) were provided by Guangdong Primate Research Center, Guangzhou, China. All animals received analgesia per-surgery. The upper, lower, left and right jaws of rhesus monkeys were selected as scaffold implantation areas (n = 16). Scaffolds were implanted into four sites of each animal to ensure that the jaw bone of each rhesus monkey was implanted with three different materials. In order to eliminate the differences between different monkeys, as well as between different regions of the jaw. Monkeys were first anesthetized with pentobarbital sodium (30 mL/kg, intravenous infusion), and the implant site was prepared with iodopovinone (Betadine) before the first and second premolars were removed. Around the extraction site, gums were separated, bone and buccal cortex were removed and intact lingual cortex was preserved, and bone defect were established (10 mm × 10 mm × 5 mm). 3D-BG, 3D-BG + BMP/CS and 3D-BG + rBMSCs + BMP/CS were inserted in contact with bone tissue to cover the damaged area, and each scaffold was implanted in different areas of the four monkeys (Supplementary Fig. S2). Lateral buccal mucosa was then sutured, and gingiva is repositioned in the opposite position. After operation, soft food was given.

### X-ray inspection

X-ray inspection of bone graft areas were taken immediately post-operation and after 12 weeks. Experimental sites of each rhesus monkey were photographed with Digital Diagnost VM X-ray machine (DR, PHILIPS, Holland).

### Micro-CT

Fresh specimens were scanned with mCT (Aloka Latheta LCT200, Japan) 12 weeks after surgery. The specimens were placed on the sample frame of the mCT system, and the continuous mCT images were obtained by scanning along the long axis of the support. The plane image resolution is 1024 × 1024. After obtaining the two-dimensional lamellar grayscale image, the 3D support model is established by software. With the help of Latheta V3.51, Mimics 15.0 and Anzlyze 12.0 software, the scanning data of the specimens were reconstructed in 3D, and the volume fraction of bone volume to tissue volume (BV/TV) was calculated.

### Histology

At 12 weeks after operation, 3% pentobarbital sodium was used for general anesthesia in rhesus monkeys and 0.5% iodophor was used to disinfect oral mucosa. Bone chisel was used to remove part of bone in experimental areas. Bone block samples were fixed in 4% paraformaldehyde, gradient dehydrated with alcohol, embedded in paraffin, and sliced using a microtome, and then stained with hematoxylin and eosin (H&E, Sigma) and Wiegert and safranin O (Sigma) working solutions respectively. The stained sections were observed with a fluorescence microscope (IX81, Olympus Corporation, Japan).

### Statistical Analysis

Samples for each group were set in quintuplicate (n = 5) in each experiment, data of one time were presented in the figures. Statistical analysis was performed in Statistical Product and Service Solutions (SPSS, 21.0). All data were initially subjected to Shapiro-Wilk normality test followed by Levene homogeneity variance test. Data were subjected to one-way Analysis of Variance (ANOVA) followed by Dunnett’s test, at 5% probability level - in case of significant F. Data with abnormal distribution were subjected to nonparametric Kruskal-Wallis test and to Dunn’s post hoc test, at 5% probability level. Difference between conditions were considered significant if p < 0.05.

## Conclusions

Repair of bone defect is a long procedure which requires a 3D biomaterial with peculiar characteristics such as porous structure and bioactivity in order to provide good conditions in terms of cell adhesion, growth space and osteogenic ability for bone tissue regeneration. 3D-BG + BMP/CS + rBMSCs was successfully prepared and characterized. Simulated human alveolar bone defects model of primate rhesus monkey was established, and the repair effect of bone defects was evaluated. Results showed that BMP/CS nanoparticles loaded on 3D-BG scaffold could promote bone regeneration ability *in vivo*, and preload of rBMSCs could promote this ability further. It indicates that the 3D-BG + BMP/CS + rBMSCs could be a promising therapeutic strategy for alveolar bone defect.

## Supplementary information

Supplementary Information

## Data Availability

Datasets generated and analysed during current study are available from the corresponding author on reasonable request.
