# Supplementary material for: Alveolar bone repair of rhesus monkeys by using BMP-2 gene and mesenchymal stem cells loaded three-dimensional printed bioglass scaffold
Source: Sci Rep. 2019 Dec 3;9:18175. doi: 10.1038/s41598-019-54551-x (PMC6890714; doi:10.1038/s41598-019-54551-x)
Supplement: Supplementary file 1 — Supplementary Information [file 41598_2019_54551_MOESM1_ESM.pdf]

**Supplemental Information to:**

# Alveolar bone repair of rhesus monkeys by using a BMP-2 gene and mesenchymal stem cells loaded three-dimensional printed bioglass scaffold

Liyan Wang[b,†], Weikang Xu[a,†], Yang Chen[c,*], Jingjing Wang[b,*]

[a]Guangdong Institute of Medical Instruments & National Engineering Research Center for Healthcare Devices, Guangzhou, Guangdong 510500, China

[b]Department of Stomatology, Foshan Woman and Children's Hospital, Foshan, Guangdong 528000, China

[c]Department of Orthopaedics, The First people's Hospital of Foshan, Foshan, Guangdong 528000, China

* Corresponding author. Department of Orthopaedics, The First people's Hospital of Foshan, Foshan, Guangdong 528000, China. Tel: +86-757-82-96-97-12; Fax: +86-757-82-96-97-12; E-mail: wangliyankmmc@163.com; and Guangdong Institute of Medical Instruments & National Engineering Research Center for Healthcare Devices, Guangzhou, Guangdong 510500, China Tel.: 086 20 39380098. E-mail addresses: 759200816@qq.com

† These authors contributed equally to this work.

## 1. Method and results of the identification of osteogenic differentiation of rBMSCs

### (1) Methods

The fourth and fifth generations of rBMSCs were seeded in 48-well plates (Costa, Corning Incorporated) at the density of $4\times10^4$ cell/well cultured in osteogenic media (OGM) for 2 weeks for studies on cell osteogenic differentiation. Alkaline phosphatase (ALP) activity on the cell layers was measured using ALP staining. Briefly, cultured cells were rinsed with PBS. And fixed in 10% neutral formalin solution for 30 min. The cells were stained using BCIP/NBT as dye for 30 min at 37 ºC, followed by washing with distilled water. Osteogenesis mineralization was assessed by alizarin red staining (ARS). Briefly, cultured cells were rinsed three times with PBS and fixed with 10% neutral formalin solution for 30 min, washed five times carefully with distilled $H_2O$ and then stained with ARS (40 mM) for 30 min at room temperature. After several washes with distilled $H_2O$ to remove excess dye, cells were examined under the optical microscope.

### (2) Results

Different day of primary culture of rBMSCs were photoed (Supplementary Fig. S2~5). After osteogenic induction of rBMSCs for 2 weeks, the cells were also photoed (Supplementary Fig. S6), and the results of ALP staining showed that there were black calcium deposits in the cells (Supplementary Fig. S1 a). Invisible white nodules can be seen with naked eye, and intracellular deposits can be stained into crimson calcium plaques by ARS (Supplementary Fig. S1b). The results of ALP and ARS showed that rBMSCs was successfully induced to differentiate into osteoblasts in vitro.

## 2. Photograph of the implanting surgery and the condition of animal after surgery

All experimental animals had no abnormal daily eating activities. The suture was absorbed by itself, and no inflammatory reaction or death was found. X-ray examinations were performed immediately post-operation to verify correct implantation of implants into bone defect area (Supplementary Fig. S7d).

**Miscellaneous Figures:**

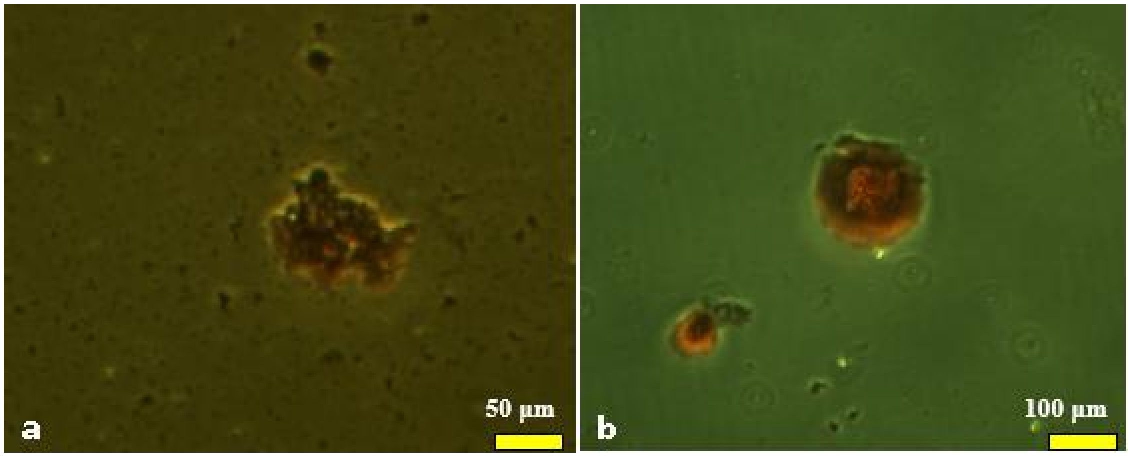


**Fig. S1** ALP staining (a) and Alizalin red staining (b) of rBMSCs after 14 days of OGM incubation.

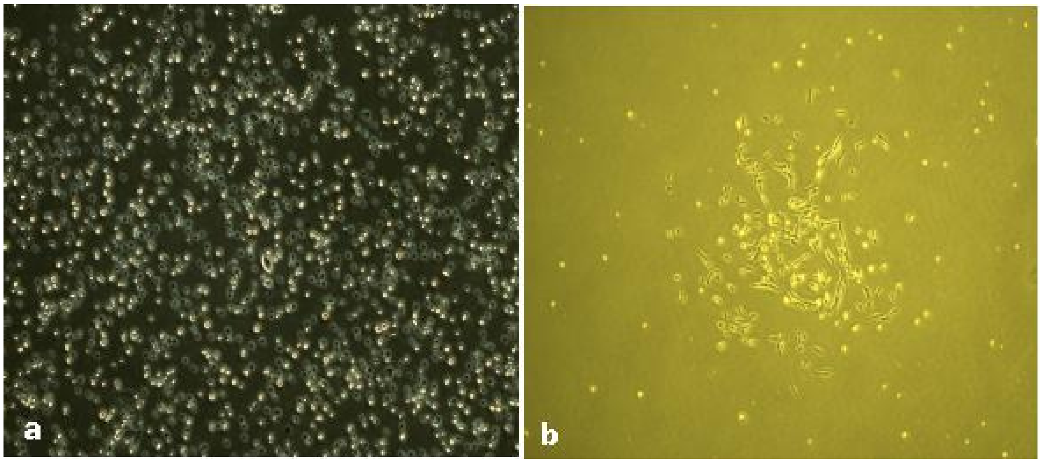

**Fig. S2** Day 1 of primary culture of rBMSCs (a, x100; b, x200). Most of the rBMSCs were suspended in the culture in a circle.

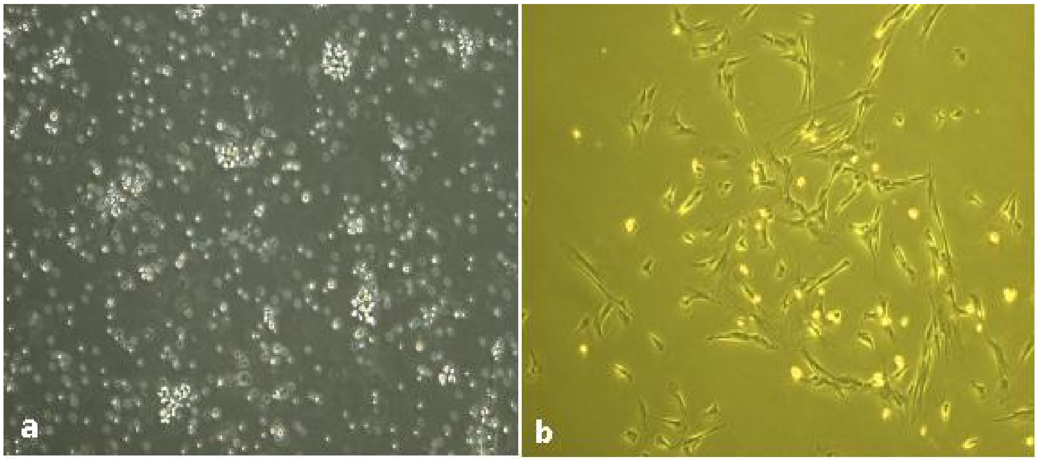


**Fig. S3** Day 5 of primary culture of rBMSCs (a, x100; b, x200). Polygonal or spindle rBMSCs were grow in small colonies.

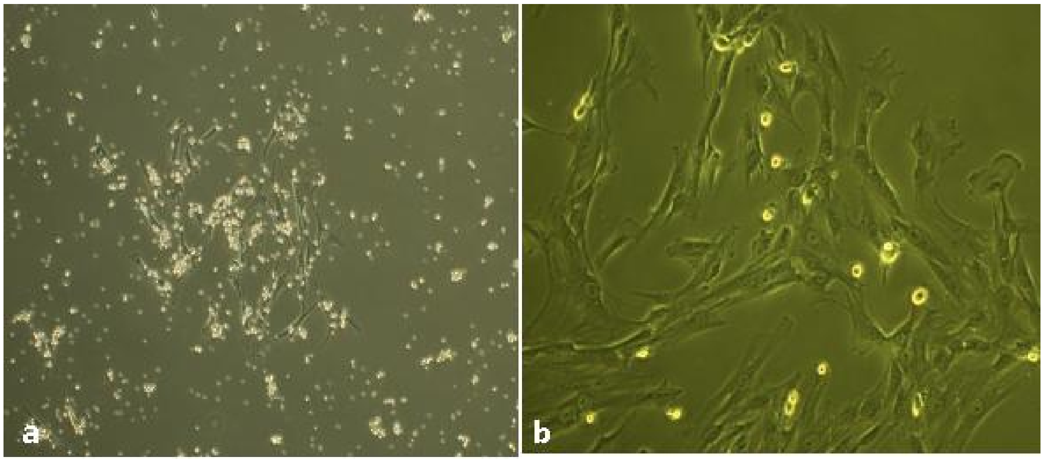


**Fig. S4** Day 8 of primary culture of rBMSCs (a, x100; b, x200). Spindle rBMSCs with large nucleus form a large number of small colonies.

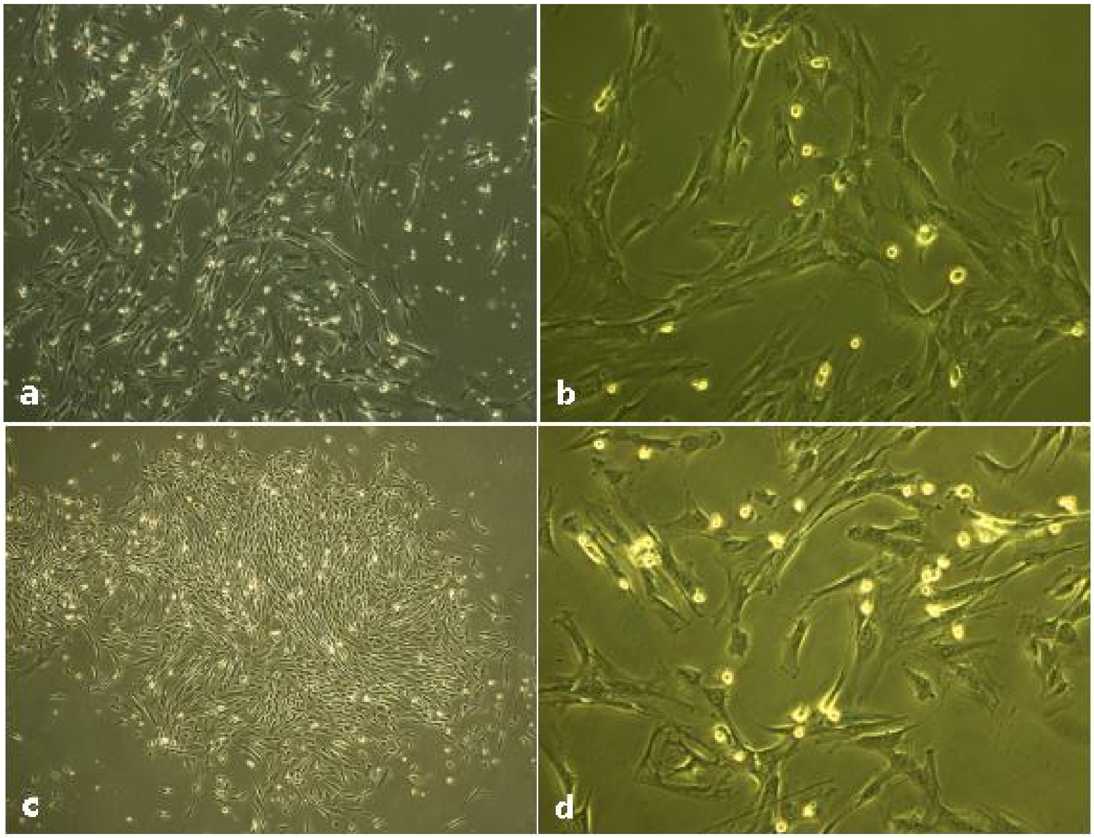

**Fig. S5** Day 10 and 14 of primary culture of rBMSCs (a,c, x100; b,d, x200). rBMSCs were colony-like growth at day 10 (a~b). Fusiform and slender rBMSCs with fibroblast-like growth were arranged in a whirlpool at day 14 (c~d).

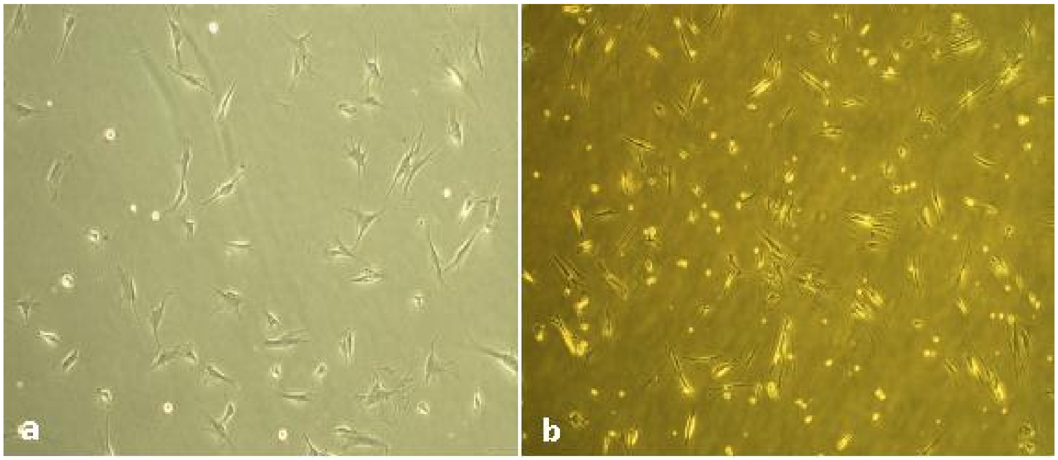

**Fig. S6** After 14 days of OGM incubation, polygonal or fusiform rBMSCs with large cell volume and rich cytoplasm were observed (a, x100; b, x200).

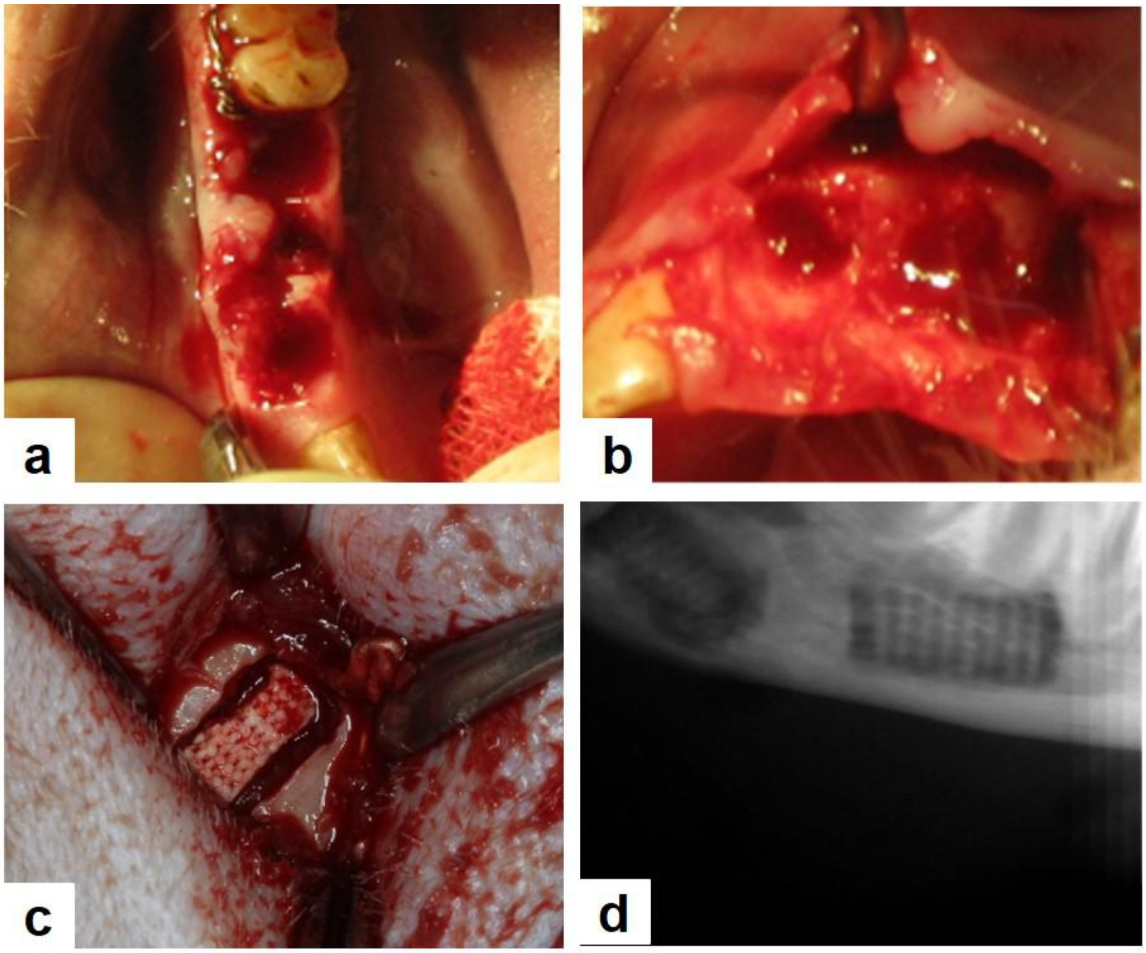


**Fig. S7** Photograph of the implanting surgery. The first and second premolar teeth were removed (a); cuboid alveolar bone defect model of 10 × 10 × 5 mm was created by removing the buccal bone cortex (b); the bone defect area filled with scaffolds (c); X-Ray examination of the scaffolds post-operation (d).
